# UAV target tracking method based on global feature interaction and anchor-frame-free perceptual feature modulation

**DOI:** 10.1371/journal.pone.0314485

**Published:** 2025-01-16

**Authors:** Yuanhong Dan, Jinyan Li, Yu Jin, Yong Ji, Zhihao Wang, Dong Cheng

**Affiliations:** Colleage of Computer Science and Engineering, Chongqing University of Technology, Chongqing, China; Jinan University, CHINA

## Abstract

Target tracking techniques in the UAV perspective utilize UAV cameras to capture video streams and identify and track specific targets in real-time. Deep learning UAV target tracking methods based on the Siamese family have achieved significant results but still face challenges regarding accuracy and speed compatibility. In this study, in order to refine the feature representation and reduce the computational effort to improve the efficiency of the tracker, we perform feature fusion in deep inter-correlation operations and introduce a global attention mechanism to enhance the model’s field of view range and feature refinement capability to improve the tracking performance for small targets. In addition, we design an anchor-free frame-aware feature modulation mechanism to reduce computation and generate high-quality anchors while optimizing the target frame refinement computation to improve the adaptability to target deformation motion. Comparison experiments with several popular algorithms on UAV tracking datasets, such as UAV123@10fps, UAV20L, and DTB70, show that the algorithm balances speed and accuracy. In order to verify the reliability of the algorithm, we built a physical experimental environment on the Jetson Orin Nano platform. We realized a real-time processing speed of 30 frames per second.

## Introduction

In recent years, unmanned aerial vehicles (UAVs) have been widely used in civil and scientific research due to their small size, easy operation, and flexibility. UAV-based target tracking integrates data processing, communication, target detection and tracking, and automatic control systems, promoting the rapid development and broad application of UAV target tracking technology. These technological advances have led to the further expansion of UAV applications in environmental detection, agricultural monitoring, and security surveillance. The main generic problem faced by UAV visual tracking in most cases is that in the operation of the actual target tracking algorithm, when the UAV flies to a certain altitude, the amplitude of the image varies considerably due to the jittering of the fuselage, resulting in blurring of the target, target occlusion, and other impacts thereby restricting the accuracy of the feature extraction and modeling, which may result in the loss of the target by the tracker. Also, in practical applications, considering the need to deploy on embedded devices, the tracker faces challenges in maintaining speed and accuracy compatibility, which becomes necessary to improve the performance of the tracker.

Among the deep learning-based target tracking methods, algorithms based on the Siamese Network framework have achieved remarkable results. Such methods mainly use Convolutional Neural Networks (CNNs) or other deep learning techniques to improve the accuracy and robustness of tracking. However, the framework suffers from two significant drawbacks. First, the cross-correlation operation is used to compute the similarity between the template image and the search region to determine the target’s location. However, this operation leads to a tracker lacking contextual information and more sensitive to scale and shape changes. The limited ability to deal with nonlinear and complex similarity relationships restricts the model’s performance in complex targets or contexts. To overcome these shortcomings, SiamAttn introduces an attention mechanism in the feature matching of twin networks, which can significantly improve the tracking performance without significantly increasing the computational effort. Specifically, SA-Siam [1] introduces an attention mechanism in the feature extraction process of twin networks, which can dynamically adjust the network’s attention to different regions and feature channels, thus improving the tracking accuracy. SiamBAN [2] combines the inter-correlation operation with an attention mechanism in the regression and classification positions and weights the feature maps by the Balanced Attention Module) Weighted feature maps, thus, better highlight the target region during matching and perform better when dealing with background clutter or occlusion. SiamGAT [3] embeds Graph Attention Network (GAT) into the Siamese framework to model the targets and neighborhood relationship. Thereby performing well in dealing with target deformation. In addition, SCSA [4] introduced the Stacked Channel-Spatial Attention (SCSA) mechanism, which was applied to visual tracking tasks to improve the efficiency and accuracy of the tracker. Therefore, it is prevalent to enhance the feature refinement representation of networks through attention, and we consider the introduction of a spatio-temporal attention mechanism that considers the global after the inter-correlation operation between the template image and the search region to improve the efficiency of the feature refinement representation of the model for nonlinear and complex cases.

In addition, anchor frames are used to generate candidate regions in image processing. However, traditional anchor frame designs often face problems of complexity, hyperparameter sensitivity, and high computational overhead, all of which affect the tracker’s real-time performance. In order to solve these problems, subsequent anchorless frame family of algorithms have been improved by generating anchor points. For example, SiamRPN [5] incorporates a region suggestion network to handle scale and shape variations better. SiamFC++ [6] uses an anchorless frame design to simplify the model design and improve tracking accuracy by directly predicting the target’s center position, width, and height without relying on predefined anchor frames. Ocean [7] network is an anchorless frame design of the Siamese network, which employs a bounding box center-based localization method for enhanced target sensing. SiamCAR [8] is an anchor-box-free Siamese network tracker that predicts the position and size of a target through two branches, classification and regression, without relying on predefined anchor boxes, thus providing higher tracking accuracy, especially in multi-scale target tracking scenarios. SiamAPN [9] introduces the Anchor Proposal Network, which aims to generate more suitable candidate regions. APN can automatically generate anchor frames that match the target’s characteristics, making these anchor frames closer to the actual target in terms of size and location, thus achieving efficient computational performance. Therefore, the anchor frame mechanism mainly affects the efficiency and complexity of the algorithm. We can enhance the training of the tracker to face multi-scale situations without affecting efficiency by combining the Anchor Proposal Network with the perceptual feature modulation, which realizes the tracking for the target deformation.

In order to solve the problems caused by the inter-correlation operation and the anchor frame mechanism and to consider both speed and compatibility, we first introduce a global attention mechanism [10] after the inter-correlation operation of the template branch and the search branch in order to enhance the global feature capturing ability of the response graph and to suppress the background noise. This enables the network to capture the salient features of the target better, thus improving the tracking accuracy in small targets and complex backgrounds. Subsequently, we designed the Anchor-Frame-Free Aware Feature Modulation (AFAFM) module. After the Anchor Proposal Network generates high-quality anchor frame drifts, we combine it with the SAFM [11] module, and by weighting the modulation of the features at each spatial location, the network is able to focus on the high-frequency information in critical regions more effectively and enhance the features, while reducing the computational burden on unimportant regions. This approach achieves multi-scale refinement in predicting the target’s location and size, simplifies the model’s complexity, enhances the adaptability to target deformation, and preserves efficiency while improving the accuracy of target localization, thus achieving the effect of compatibility between efficiency and speed.

The main contributions can be summarized as follows:

- A novel UAV target tracking method is proposed under a deep learning tracking framework based on the Siamese family. This framework combines global feature interaction and anchor-free frame-aware feature modulation techniques.
- A Global Attention Mechanism (GAM) [10] module is introduced, which enhances the global feature capture capability of the response map by feature refinement of the response map generated by the inter-correlation operation between the template frame and the search region. This improvement optimizes the feature representation of the response map and enables the model to perform target tracking more effectively when facing tiny targets and complex scenes. Meanwhile, the anchor-free frame-aware feature modulation (AFAFM) module is designed to perform spatial position-weighted modulation after the drift of high-quality anchor frames. This makes the features of the candidate frames more prominent, enhancing the difference between the target and the background and better adapting to the target’s shape and scale changes.
- Comparisons with other tracking methods are made on UAV vision datasets such as UAV123, UAV20L, and DTB70, verifying our algorithm’s advantages in terms of accuracy and speed. Meanwhile, a physical platform was built to verify the algorithm’s practical feasibility and ensure that the speed requirements in real applications are met.

This study designed a new approach to address the above challenges. First, a global attention mechanism (GAM) is introduced to enhance feature representation by considering spatial and channel information. In addition, we design the anchor-frame-free perceptual feature modulation (AFFPFM) module, which performs feature optimization and improves target localization accuracy by modulating the feature map at multiple scales. Finally, to verify the practicality of the algorithm, we built a physical platform and verified the algorithm on a real UAV, which successfully realized the tracking of the target.

## Relate workers

Single-target tracking algorithms are mainly categorized into two main branches: tracking algorithms based on correlation filtering and tracking algorithms based on deep learning. This study focuses on the Siamese family of deep learning-based frameworks and, therefore, will focus on the Siamese family of related methods.

The correlation filtering algorithm significantly improves the tracking speed by converting the solution process to the frequency domain, which enables the algorithm to realize the real-time tracking effect. However, the core challenge of the correlated filter tracking algorithm is solving the filter efficiently. In 2010, Bolme et al. introduced the correlated filter algorithm into target tracking for the first time and proposed the Minimum Output Sum of Squared Error (MOSSE) tracking algorithm [12]. Subsequently, Danelljan et al. proposed the DSST algorithm [13], which deals with large-scale variations in complex image sequences through a robust scale estimation method. Henriques et al. proposed the kernelized correlation filter (KCF) [14], which reduces the amount of storage and computation through the discrete Fourier transform to improve the speed further. The DCF correlation tracking algorithm introduces multi-channel features (e.g., HOG features) to improve the tracking performance further. Yiming et al. proposed the AutoTrack algorithm [15], which introduces an online automatic adaptive learning of spatio-temporal regularization based on the DCF algorithm, using the spatial local response map variation as the spatial regularization. SRDCF [16] proposes a method to optimize the correlation filter using the spatial regularization technique, which can effectively suppress background interference and adapt to the target scale changes. STRCF [17] extended the SRDCF to improve the filter’s ability to deal with the target appearance changes and motion uncertainty by applying regularization in spatial and temporal dimensions. DI YUAN et al., based on the DCF filter, proposed ASTCA [18], which more comprehensively characterizes the dynamic changes of the target during the tracking process by simultaneously considering the spatial and temporal characteristics of the target. SCSTCF [19] is a target-tracking algorithm based on correlation filtering, which improves the tracking effect by enhancing the spatial and channel selection capabilities of the correlation filter and introducing a temporal regularization. Zhang et al. [20] introduced sparsity and spatial regularization mechanisms to enhance the tracking performance of correlation filters further. The main advantage of such algorithms is that they are real-time usually require only CPU support, and are easy to deploy on embedded devices. However, relative to deep learning target tracking algorithms, correlation filtering algorithms usually use HOG or grayscale features, which have a limited ability to represent complex objects and make it challenging to capture the deep features of the target adequately. The problem of target loss is more common in complex situations such as illumination change, scale change, fast motion, and severe occlusion, and the robustness of the model is relatively poor. Thus, there is a gap in precision and accuracy compared to deep learning methods.

In addition, there are a number of studies combining filters with deep learning algorithms. For example, self-SDCT [21] combines deep learning and correlation filtering to train a deep neural network through self-supervised learning and utilizes correlation filters for efficient target tracking. Another example is Recapture-SiamCF [22], which combines the traditional correlation filtering method and the Siamese network structure, synthesizing the advantages of both. The recapture mechanism further improves tracking stability and accuracy.

On the other hand, lightweight twin networks of the Siamese family occupy an important place in deep learning-based target-tracking algorithms. Bertinetto et al. proposed the SiamFC algorithm [23], which learns the similarity of targets by training a fully convolutional twin network. The network uses a fully convolutional architecture and inter-correlation operations and performs target tracking through template matching in the inference phase, validating the great potential of Siamese family-based trackers. Thus, an efficient Siamese-based tracker is also a promising option for UAV tracking.

Under the branch of Siamese networks, Siamese-based attention networks were first actively explored to enhance the expressive ability of feature refinement. SiamAttn incorporated attention mechanisms to improve the adaptive ability to changes in complex backgrounds and target appearance through finer-grained feature selection. SA-Siam [1] used spatial attention and Channel Attention mechanisms to enhance the network’s ability to discriminate between targets and backgrounds. SiamBAN [2] incorporates Adaptive Attention Mechanisms into a twin network and uses an anchorless frame design to improve its ability to handle changes in target scale. SiamGAT [24] introduces Graph Attention Mechanisms (GAMs) to enhance its ability to adapt to changes in target appearance. Attention Mechanism (Graph Attention Mechanism) captures complex relationships between targets and backgrounds by building graph structures on feature graphs. As a result, the Attention Mechanism plays different roles at the module positions in each branch. In addition, transformer structures have even been introduced. For example, CorrFormer [25] combines the Transformer structure with the cross-correlation operation to enhance the context-awareness capability through the Transformer and utilizes cross-correlation for target matching and tracking. SCATT [26] proposes a Transformer-based target tracking algorithm, which combines the symmetric cross-attention mechanism that fully utilizes the advantages of the Transformer and enhances the context-awareness capability.

Next, for the anchor frame mechanism in the Siamese family, SiamRPN introduces a region proposal network (RPN) based on SiamFC to generate multiple candidate frames, which enhances the target localization accuracy, especially in complex backgrounds. DaSiamRPN [27] employs the anchor point mechanism based on SiamRPN and extends the negative sample generation strategy to improve the robustness to background interference and appearance changes. SiamRPN++ [28] improved SiamRPN by using deep features and introducing multi-scale anchors and multi-scale feature fusion, improving tracking accuracy and robustness. SiamMask [29] added a saliency detection module based on SiamRPN, which is capable of outputting the target’s accurate segmentation mask, which realizes the unification of target segmentation and tracking. SiamFC++ [6] incorporates Anchor-free Design (AFD), which simplifies the network structure and improves the flexibility and accuracy of localization by directly regressing to the target centroid and bounding box. SiamCAR [8] simplifies the design of SiamRPN by using Anchor-free classification and regression branching instead of RPN, which reduces the complexity associated with anchor point design and provides more efficient and accurate target tracking. SiamAPN [9] introduces an Adaptive Proposition Network (APN) module to predict the target size and shape better, enhancing the tracking performance in complex scenarios. Therefore, the choice of anchor frame mechanism and the way target location determination is performed after generating anchors affects the accuracy of the classification and regression results, affecting the tracker’s efficiency and speed.

In summary, considering the location of the attention mechanism and the design of the anchor frame structure, we propose an improved UAV target tracking method based on global feature interaction with anchor frame-free perceptual feature modulation. First, we introduce the global attention mechanism (GAM) after the necessary operations of the twin network to interrelate the template frames and search branches. By combining temporal attention and channel attention, GAM can enhance the global feature-capturing ability of the response map and optimize the feature representation so that the model can track the target more efficiently in the face of tiny targets and complex scenes. In addition, we design the Anchor-Frame-Free Aware Feature Modulation (AFAFM) module. This module performs spatial position-weighted modulation after the drift of high-quality anchor frames to make the features of candidate frames more prominent. This method not only enhances the difference between the target and the background but also better adapts to the shape and scale variations of the target, thus improving the tracking accuracy and robustness.

## Global feature interaction with anchorless frame perception feature modulation UAV tracking algorithm

### Overall algorithm

**Fig 1:**
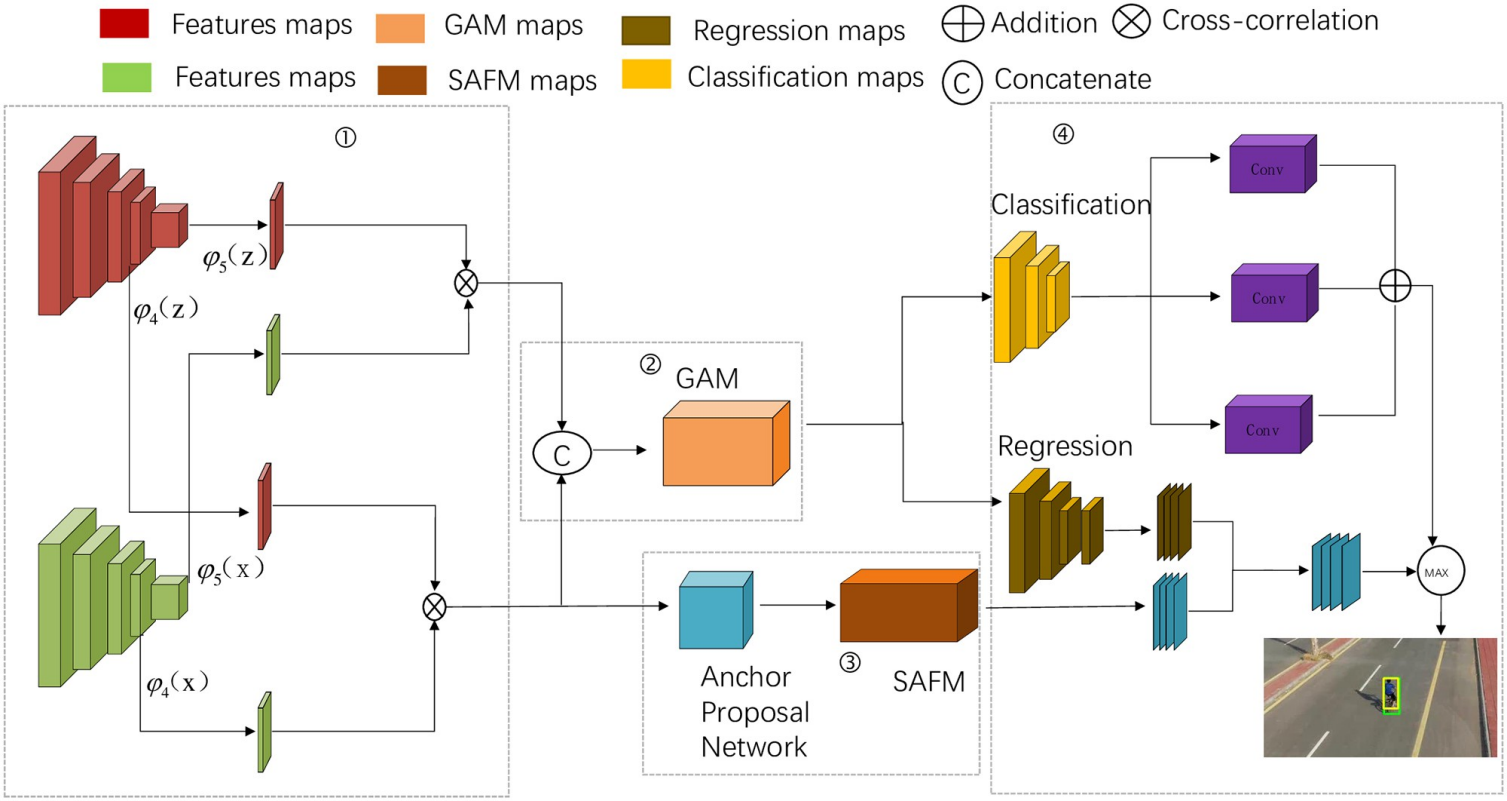
The overview of the GAFFPFM tracker. It composes of four subnetworks, i.e., feature extraction network, feature fusion network, anchor-frame-free perceptual feature modulation (AFFPFM), and multi-classification regression network.

This study proposes a UAV target tracking method based on global feature interaction with anchor frame-free perceptual feature modulation to address the above challenges. The general twin network-based target tracking algorithm mainly includes the input of the template frame and target frame, feature modeling, target localization, and the final output of the predicted position of the target frame; the flow of the algorithm is shown in Fig 1 is specifically divided into the following four parts:

(1) Feature extraction part: a twin network framework using AlexNet as the backbone network is used. At the same time, the hierarchical feature extraction between the fourth and fifth layers is performed, and the fifth layer features of the template image are inter-correlated with the fifth layer features of the search image for mutual correlation operation, which is entered into the feature fusion network. At the same time, the fourth layer features of the template image are fused with the fourth layer features of the search image by mutual correlation and entered into the SAPN network to improve the feature expression ability of the model.
(2) Feature fusion part: after the inter-correlation operation of the fourth and fifth layer network feature maps, the connection operation of the feature maps is carried out. The GAM module is introduced to dynamically adjust the importance of each channel to enhance the quality of feature representation, which helps better capture the critical information of the input data and provide robust information for subsequent target classification and regression branching.
(3) AFFPFM network: The anchor-frame-free perceptual feature modulation (AFFPFM) module adopts the anchor frame-free mechanism, which significantly improves the speed of target search. Meanwhile, the SAFM module is introduced to enhance the feature representation. This module further improves the feature expressiveness by modulating the features at multiple scales, thus realizing more accurate target localization.
(4) Classification and regression network: appropriate loss functions are constructed through a three-layer classification network and a two-layer regression feature fusion network to enhance the classification and regression capabilities of the target and generate accurate prediction frames.

First, the feature extraction network uses AlexNet as the backbone and adopts the classical twin network structure. One branch receives the template image as input, and the other receives the search target image as input. The feature maps of the fourth and fifth layers are extracted simultaneously after five layers of convolution. The output of the upper layer template branch is *φ*_4_(*z*), *φ*_5_(*z*) and the output of the search target branch is *φ*_4_(*x*), *φ*_5_(*x*). Next, a mutual correlation operation is performed on *φ*_4_(*z*), *φ*_4_(*x*) and *φ*_5_(*z*), *φ*_5_(*x*) to obtain the response graphs *R*_1_, *R*_2_, which are then joined by a join operation. The connected feature map is input to the channel-space interaction module (GAM). This module is designed to enhance the expressive power of the template features, adjust the contextual information, and strengthen the relationship between global and local features to reduce information dispersion effectively. This structural design can more fully utilize the twin network structure of AlexNet to enhance the target tracking algorithm’s feature expression capability and accuracy through the mutual correlation operation and the channel-space interaction module.

$$\begin{array}{c} R_{1}=c\left(\varphi_{4}\left(x\right)\right)⊗c\left(\varphi_{4}\left(z\right)\right) \end{array}$$ (1)

$$\begin{array}{c} R_{2}=c\left(\varphi_{5}\left(x\right)\right)⊗c\left(\varphi_{5}\left(z\right)\right) \end{array}$$ (2)

The *c*() in Eqs (1) and (2) denote the corresponding different convolution operations, and *R*_1_, *R*_2_ represent different response maps, respectively. After the deep inter-correlation operations *R*_1_, *R*_2_, a global attention enhancement mechanism is introduced to improve the effectiveness of the feature fusion network. Next, *R*_1_ is input to the anchorless frame mechanism network, while the SAFM network with multi-scale features is introduced. The SAFM network improves the resolution of the features without affecting the efficiency of the anchorless frame mechanism network and provides robust information for target regression. Finally, the outputs of the fused spatial and channel feature information and the anchorless frame-aware feature modulation network are fed into the target’s classification and regression networks, respectively, to determine the target’s location to be tracked.

### GAM global attention mechanism

In single-target tracking algorithms based on the Siamese family, a mutual correlation operation is used to compute the similarity between the template image and the search region to determine the location of the target. However, this approach results in a tracker that lacks contextual information, is sensitive to scale and shape variations, and has limited ability to deal with nonlinear and complex similarity relationships, thus limiting the model’s performance when dealing with complex targets or backgrounds. To overcome these problems, we introduce the Global Attention Mechanism (GAM) [10], which is an enhanced version of the CBMA module [30] capable of capturing global information. After the inter-correlation operation, the GAM mechanism is used to perform feature refinement, enhance the global feature capture capability of the response map, and optimize the feature representation, which enables the model to perform target tracking more efficiently when confronted with tiny targets and complex scenes. Rahman [4] et al. improved the tracking performance by combining the CBMA module with the SiamFC fusion, which enhances the tracking effect but reduces the tracking speed of the algorithm. Therefore, to improve the performance of the tracking algorithm, we introduced the Global Attention Mechanism (GAM) module, specifically designed to enhance cross-dimensional interactions between features [9]. This module provides vital information for subsequent classification and regression tasks. The Global Attention Mechanism enhances the interaction between channels and spatial information by dynamically adjusting the importance of each channel in the feature map. It captures more global contextual information and efficiently selects and emphasizes important features, thereby reducing information loss and improving gradient propagation. This approach significantly improves the model’s feature representation and overall performance.

**Fig 2:**
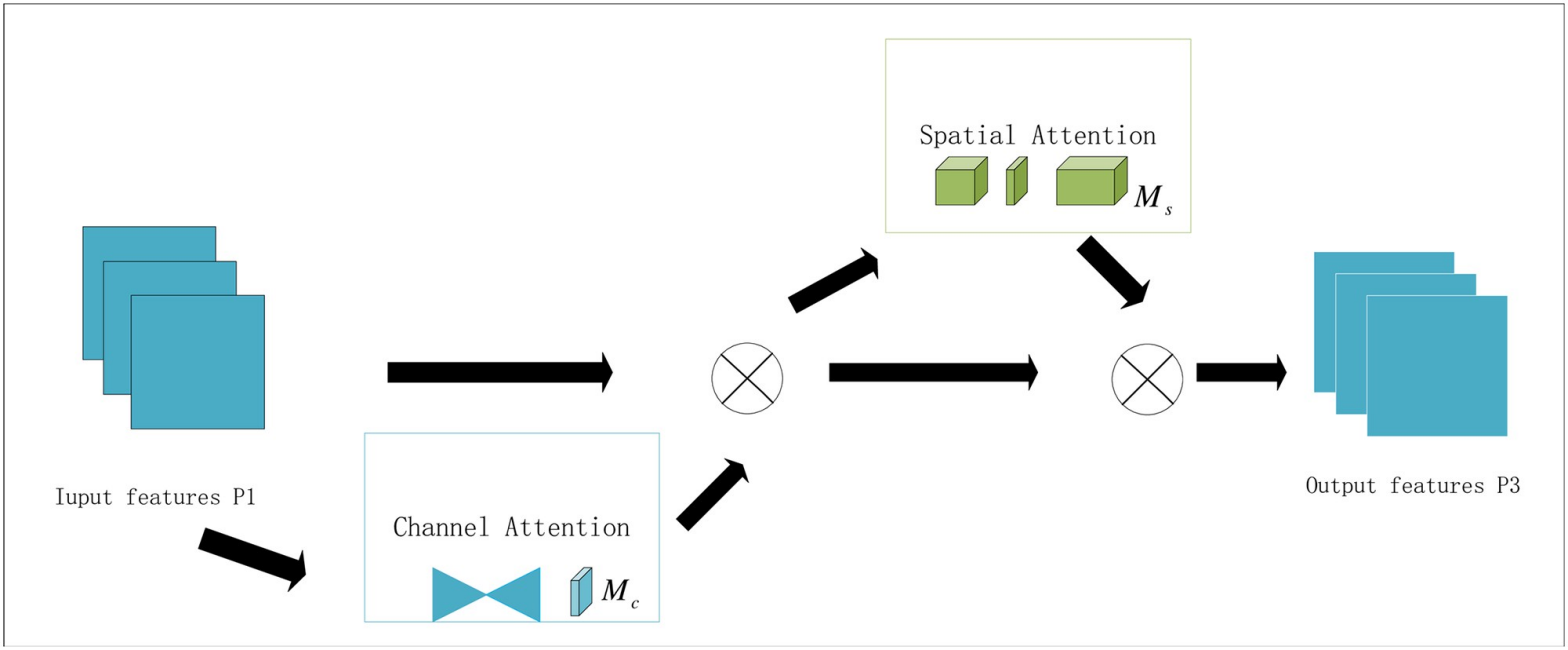
The overview of GAM. Channel attention and spatial attention are included.

Specifically, the spatial and channel attention modules are shown in Fig 2, which illustrates a schematic diagram for enhancing cross-dimensional interactions by preserving channel and spatial information to improve the performance of deep neural networks. In this figure, the Global Attention Mechanism (GAM) enhances the performance of the image categorization task by introducing 3D alignment and Multi-Layer Perceptron (MLP) for channel attention in conjunction with the Convolutional Spatial Attention sub-module. The introduction of 3D alignment and MLP for channel attention dynamically adjusts the importance of each channel in the feature map to enhance the channel dependency between features. On the other hand, the convolutional spatial attention sub-module helps to capture and improve the spatial structures in the feature maps, which are particularly critical when dealing with image classification tasks. This combined use of channel and spatial attention allows the model to capture global contextual information more efficiently and optimize the selection and emphasis of essential features, thus improving the performance of deep neural networks in complex tasks.

$$\begin{array}{c} P_{2}=M_{c}\left(P_{1}\right)⊗P_{1} \end{array}$$ (3)

$$\begin{array}{c} P_{3}=M_{s}\left(P_{2}\right)⊗P_{2} \end{array}$$ (4)

In Eqs (3) and (4), *M*_*c*_ and *M*_*s*_ denote the channel and spatial attention maps, respectively, and denote the elements that undergo a multiplication operation. This operation applies channel attention weights to each channel of the input feature map to enhance the representation of essential features. The *P*_1_, *P*_2_, *P*_3_ denote the input feature maps, the feature maps generated by the channel attention module, and the feature maps generated by the spatial channel module, respectively. This operation applies spatial attention weights to each spatial location of the input feature map to enhance the representation of specific spatial regions.

**Fig 3:**
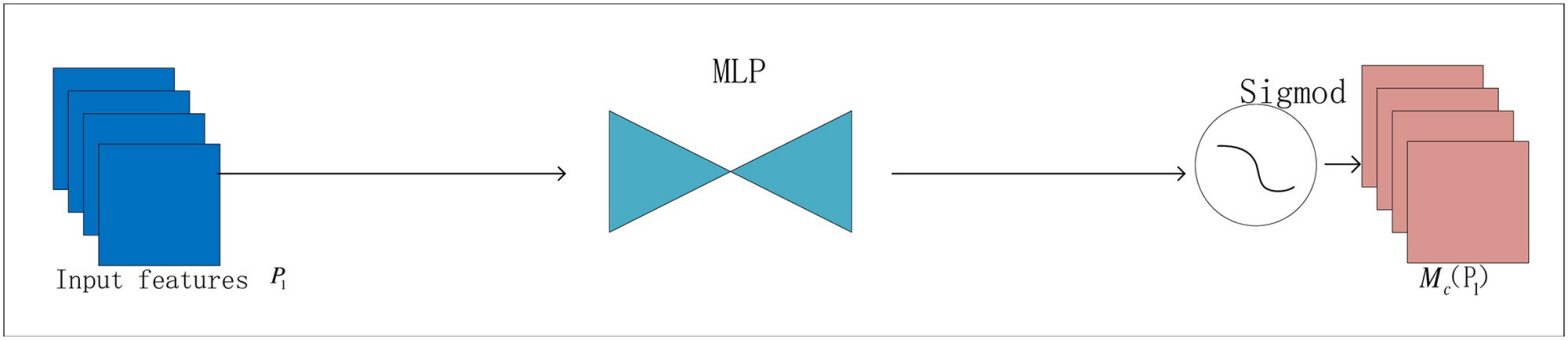
Channel attention module.

Fig 3 shows the channel attention module. It uses a three-dimensional arrangement to preserve three-dimensional information and then a two-layer multilayer perceptron (MLP) with powerful function approximation to amplify the channel-space correlation across dimensions, generating a channel attention feature map.

**Fig 4:**
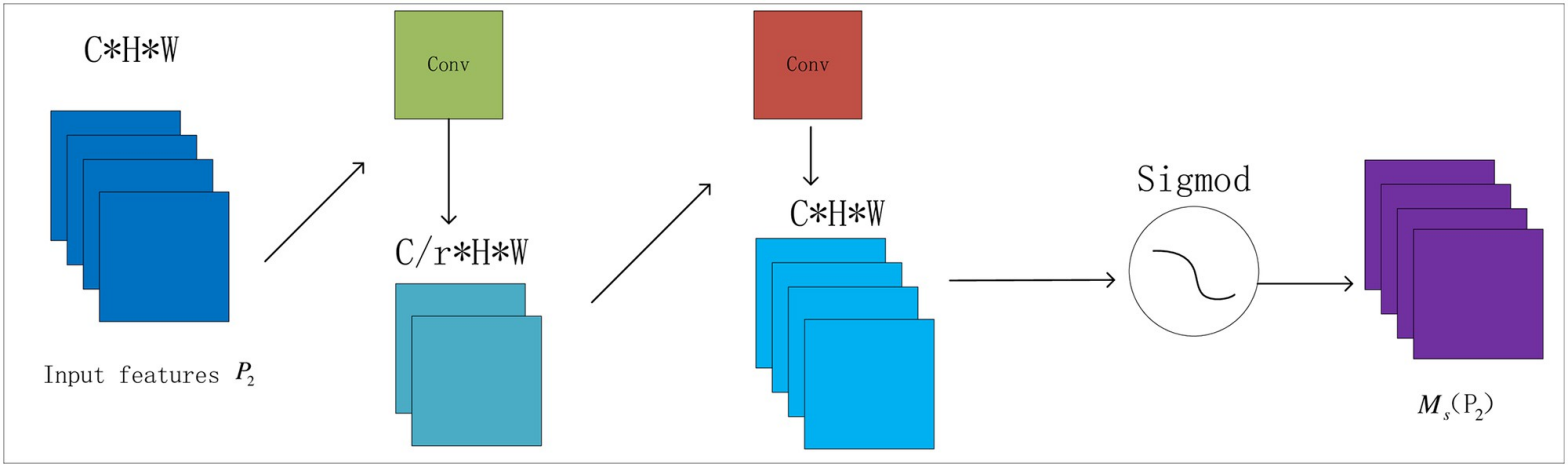
Spatial attention module.

The spatial attention module is shown in Fig 4. In the spatial attention sub-module, two convolution layers are used for spatial feature fusion to centralize the spatial information. To effectively integrate features, improve reconstruction performance, and enhance cross-dimensional interactions, we adopt the same channel attention module as BAM and set the reduction rate r to reduce the number of intermediate-layer feature maps, thus reducing the computation and storage requirements. We avoid pooling operations to preserve feature mapping, further, accelerate model convergence through dimensionality reduction and normalization, and finally generate spatial attention maps for weighting.

### Anchorless frame mechanism

For target tracking algorithms in UAV scenarios, lightweight and adaptivity not only improve the tracking stability under the UAV perspective but also meet the real-time requirements of the algorithms. The anchor frame mechanism in this paper generates an anchor frame only at each position in the similarity map and, after the response map, generates an anchor point (*i*, *j*) at each of its positions. It corresponds to searching for the x-image under the (*p*_*i*_, *p*_*j*_).

$$\begin{array}{c} p_{i}=\frac{w_{s}}{2}+\left(i-\frac{w}{2}\right)\times s \end{array}$$ (5)

$$\begin{array}{c} p_{j}=\frac{h_{s}}{2}+\left(j-\frac{h}{2}\right)\times s \end{array}$$ (6)

In Eqs (5) and (6), *w*_*s*_ and *h*_*s*_ denote the width and height of the searched image, respectively, and s is the step size of the network. Additional classification and regression branches are introduced to determine the location and size of the target frame. The classification and regression branches are induced from the response graph R. The classification branch outputs three classification feature maps: $H_{w\times h\times 2}^{cls1},H_{w\times h\times 2}^{cls2},H_{w\times h\times 2}^{cls3}$, $H_{w\times h\times 2}^{cls1}$ each point (*w*, *h*,), is used to indicate the positivity or negativity of each anchor point, in the ratio to the real, we set the threshold to 0.7, when greater than 0.7 is a positive case anchor, and less than 0.7 is a negative case anchor, and the two-dimensional vectors in $H_{w\times h\times 2}^{cls2}$ denote the foreground and background in the search image x’s fractions, which are foreground when falling within the true frame and background otherwise. The $H_{w\times h\times 2}^{cls3}$ denotes the mass of each frame:

$$\begin{array}{c} l=x-x_{0},t=y-y_{0},r=x_{1}-x,b=y_{1}-y \end{array}$$ (7)

As in Eq (7) above, are the positions corresponding to the corresponding locations in the search image to the four edges of the bounding box, where (*x*_0_, *y*_0_), (*x*_1_, *y*_1_) are the coordinates of the upper-left corner and the lower-right corner of the real box, and (*x*, *y*) are the positions corresponding to the images generated by our anchor point (*i*, *j*), So, to summarize, the total classification loss function [9] can be found as Eq (8):

$$\begin{array}{c} L_{cls}=\lambda_{cls1}L_{cls1}+\lambda_{cls2}L_{cls2}+\lambda_{cls3}L_{cls3} \end{array}$$ (8)

In addition, the response map with the feature maps of the branches of the regression: $H_{w\times h\times 4}^{loc1}$ and $H_{w\times h\times 4}^{loc2}$, which are outputted in the response map, indicates that the distance of the searching image *x*, *y* to the borders of our actual image can be expressed by the above Eq (7), while the distance of the searching image to the borders of the actual image corresponding to $H_{w\times h\times 4}^{loc2}$ of the search image to the actual image border distance can be expressed as the following equation:

$$\begin{array}{c} {{\tilde{r}}^{0}}_{\left(i,j\right)}=\frac{g_{x}-p_{x}}{p_{x}},{{\tilde{r}}^{1}}_{\left(i,j\right)}=\frac{g_{y}-p_{y}}{p_{y}},{{\tilde{r}}^{2}}_{\left(i,j\right)}=\text{ln}\frac{g_{w}}{p_{w}},{{\tilde{r}}^{3}}_{\left(i,j\right)}=\text{ln}\frac{g_{h}}{p_{h}} \end{array}$$ (9)

where in Eq (9), *g*_*x*_, *g*_*y*_, *g*_*w*_, *g*_*h*_ denote the center point coordinates of the true coordinates and the width and height of the box, respectively, and *p*_*x*_, *p*_*y*_, *p*_*w*_, *p*_*h*_ denote the center point coordinates of the predicted coordinates and the width and height of the box, respectively. This results in an overall regression loss function [9] of:

$$\begin{array}{c} L_{loc}=\lambda_{loc1}L_{loc1}+\lambda_{loc2}L_{loc2} \end{array}$$ (10)

In summary, the overall loss function is:

$$\begin{array}{c} L=\lambda_{1}L_{cls}+\lambda_{2}L_{loc} \end{array}$$ (11)

### Anchor-frame-free perceptual feature modulation

SAFM (Spatially-Adaptive Feature Modulation) was originally used to independently computationally learn multi-scale feature representations and dynamically aggregate these features for spatial modulation [11], which is mainly applied to image super-resolution tasks. In this paper, an anchor-free frame-aware feature modulation module is designed to perform spatial location-weighted modulation after the drift of high-quality anchor frames, which can make the features of these candidate frames more prominent, not only to enhance the difference between the target and the background but also to better adapt to the target’s shape and scale changes.

In this paper, we design the SAFM module to accept feature maps generated by anchorless frames as input. By taking the feature maps as input, the SAFM module can utilize the information extracted by the neural network more efficiently, thus improving the performance and efficiency of the super-resolution task. We explore the mechanism of remote adaptation based on multi-scale feature modulation of the anchorless frame feature maps. To enhance the local context information, we further develop a compact method that improves the feature expressiveness and detail-capturing ability while effectively utilizing the multi-scale feature information, reducing the computational complexity, and significantly improving the features’ quality and the overall network’s efficiency.

The FMM module consists of SAFM and CCM to select features. Thus, the network can be referred to as a unified feature mixing module, thereby selecting representative features that can be represented as:

$$\begin{array}{c} Y=SAFM(LN(X))+X \end{array}$$ (12)

$$\begin{array}{c} Z=CCM(LN(Y))+Y \end{array}$$ (13)

Where *LN* is the LayerNorm layer in Eqs (12) and (13), and is the variable generated by the intermediate transformation.

**Fig 5:**
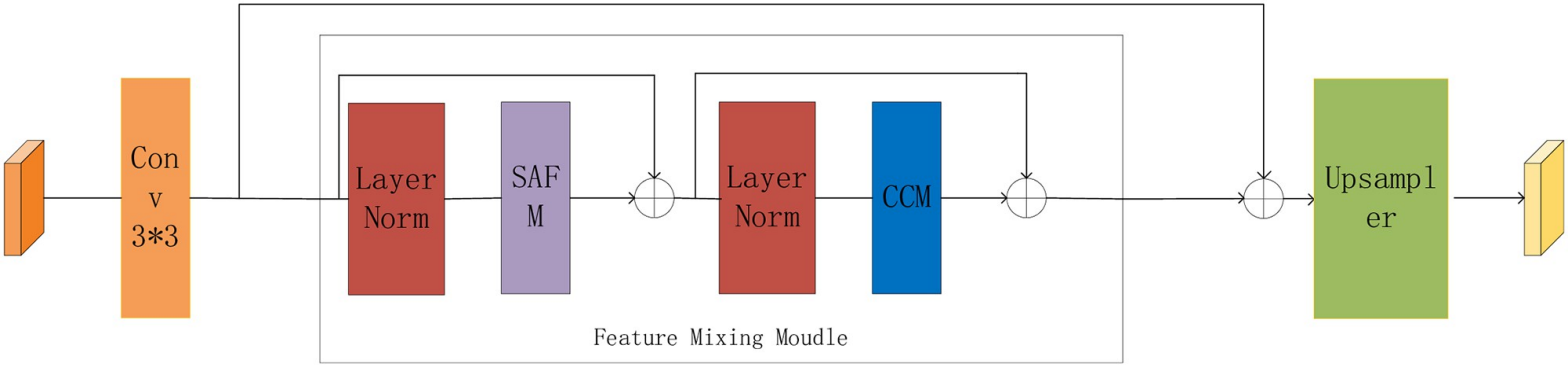
An overview of the proposed SAFM. Mainly includes the FFM module.

As shown in Fig 5 above, the network mainly consists of a Functional Mixing Module (FMM) and an upsampler layer:

- A 3 × 3 convolutional layer transforms the input feature map to generate shallow features.
- These features are fed into the FMM for high-resolution feature map reconstruction. The FMM consists of a spatial adaptive feature mechanism (SAFM) and a convolutional channel mixer (CMM), while global residuals are introduced to learn high-frequency details.
- A lightweight upsampling layer performs fast reconstruction.

Under the SAFM module, the features are divided into four different scales. Each scale is processed with varying depths of convolution, and then features are aggregated by 1 × 1 convolutional layers. Then, the ReLU activation function is applied to realize feature modulation. The feature with scale 0 is processed by deep convolution, while the remaining three scales are reduced in feature resolution by adaptive maximum pooling. Deep convolution is then applied again, and features are sampled back to their original size using nearest neighbor interpolation. Finally, the training process is normalized by a normalization layer. In CCM, local information is fused by enhancing the local spatial modeling capability by first encoding the spatial local context using a 3 × 3 convolution and then reducing the number of channels back to the original input dimension by a 1 × 1 convolution.

$$\begin{array}{c} F_{0}=C\left(F_{e}\right) \end{array}$$ (14)

$$\begin{array}{c} F_{re}=℧\left(M_{\theta }\left(F_{0}\right)+F_{0}\right) \end{array}$$ (15)

In Eqs (14) and (15), *F*_0_ represents the shallow features after convolution, *F*_*e*_ represents the input features, *F*_*e*_ and then the FMM module, ℧ represents the upsampler function, and *M*_*θ*_ represents the FFM processing, which generates the feature map after *F*_*re*_ reshaping.

$$\begin{array}{c} L=\|F_{re}-F_{r}{\|}_{1}+\lambda \|f\left(F_{re}\right)-f\left(F_{r}\right){\|}_{1} \end{array}$$ (16)

where in Eq (16), *f* represents the Fourier function transform, *F*_*r*_ is the high-quality real feature map, and *F*_*re*_ is the post-generation remodeled feature map.

## Experimental analysis

### Implementation details and evaluation criterion

#### Implementation details

The experimental platform for this algorithm was configured as follows: a 12th Gen Intel(R) Core(TM) i7-12700K CPU with a GTX 3080 Ti graphics card was used, and the operating system was Ubuntu 18.04. The algorithm’s implementation was based on PyTorch 1.10 under Python version 3.6. The source of the training data was the COCO [31] dataset, which contains 91 classes of targets and 2.5 million labels.

The network uses Alexnet as the backbone; the parameters of the first two convolutional layers were frozen and loaded with pre-trained models, the last three convolutional layers were fine-tuned, and the feature extraction network was frozen for the first ten epochs. The anchor-less frame mechanism network was trained, the feature fusion network, the feature augmentation, and the spatio-temporal augmentation network, and the last 30 epochs were implemented to train the whole network end-to-end, and the learning rate was reduced from 0.005 to 0.0005 with a momentum of 0.9. At the same time, the template and search image sizes are 127*127 and 287*287 pixels, respectively. Meanwhile, taking into account the short and efficient training, as well as the comparison of training accuracy, we are taking to compare P and S under the same conditions; using the same dataset on a BASELINE is a common and effective way to evaluate the performance, to ensure the consistency of the data, and to ensure the same training set, as well as the test set, i.e., the demonstrated SiamAPN as well as our algorithms, were trained under the coco dataset to perform the accuracy comparison.

#### Evaluation criterion

In order to fully evaluate the performance of our algorithm, we use the following evaluation metrics:

1. Precision of OPE: One-pass Evaluation (OPE) method is used for evaluation. We initialize the first frame at the position of the target in ground-truth, and after running the tracking algorithm, we calculate the center position error between the predicted frame and the real frame. Accuracy is measured by calculating the proportion of frames where the center error is less than a specified threshold (e.g., 20 pixels).
2. Success Plot of OPE: The success rate is calculated based on the Intersection over Union (IoU) between the predicted frame and the real frame. The success rate represents the proportion of frames with IoU greater than or equal to a specified threshold. By plotting the success rate, the performance of the algorithm under different IoU thresholds can be visualized.
3. Frames Per Second (FPS): FPS measures the number of frames processed per second by the algorithm. A higher frame processing speed means that the algorithm is more real-time and thus better able to meet real-time tracking requirements.

### State-of-the-Art comparison

#### Experiment on UAV123@10fps dataset

**Fig 6:**
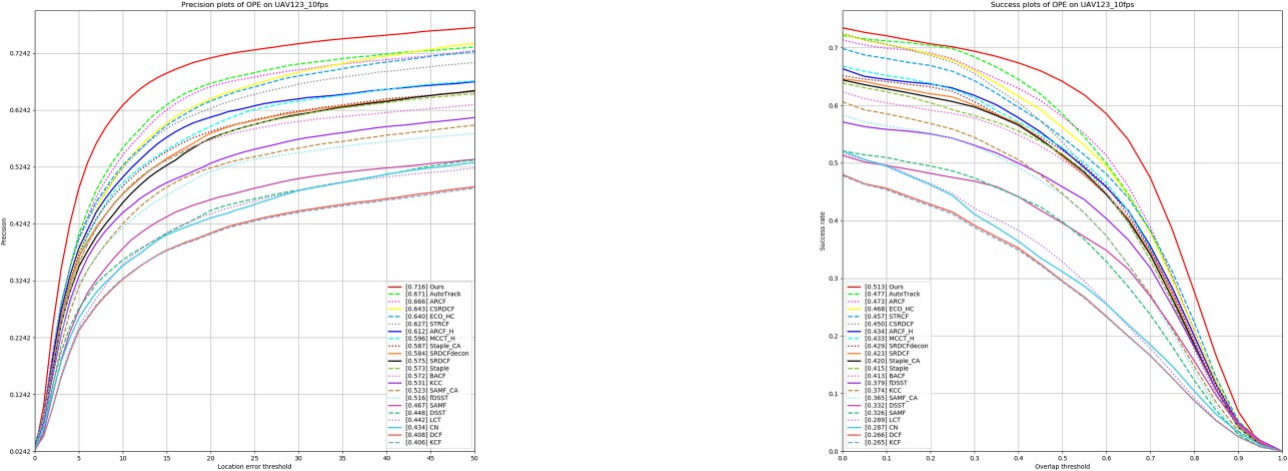
Experimental results on the UAV123@10fps dataset.

The UAV123@10FPS dataset contains 123 sequences, each captured at ten frames per second. This adds multiple challenges due to the large scene variations between frames, including fast motion, low resolution, aspect ratio variations, background clutter, fast camera movement, motion blur, and occlusion. Therefore, the UAV123@10FPS is more suitable for evaluating tracker performance than the UAV123 dataset. This dataset is suitable for evaluating the performance of the tracker in UAV low frame rate videos, especially the performance of the target in relatively stable or slow-moving scenes. To validate the tracking effectiveness of our algorithm GAFFPFM, as shown in Table 1 and Fig 6, we compare it with 25 other trackers, including SiamAPN [9], TADT [32], SiamFC [23], UDT [33], AutoTrack [15], ARCF [34], ARCF_F [34], CSRDCF [35], ECO_HC [36], STRCF [17], MCCT_H [37], Staple [38], Staple_CA [38], SRDCF_decon [39], SRDCF [16], BACF [40], KCC, SAMF [41], SAMF_CA [41], fDSST [42], DSST [43], LCT [44], CN, DCF, and KCF [14]. These comparisons include classical deep learning algorithms (e.g., SiamAPN, SiamFC, UDT, AutoTrack, etc.) as well as classical filter-like tracking algorithms (e.g., ARCF, SRDCF, BACF, STRCF, KCF, etc.). The results demonstrate the superior performance of our tracker in terms of precision (Precision) and area under the curve (AUC). Compared with the baseline algorithm SiamAPN, our Precision score improves by 2.6%, and our AUC score improves by 3.4%, which fully demonstrates our advantages in feature refinement and perceptual modulation.

**Table 1:** Experimental results on the UAV123@10fps dataset of precision scores, success scores.

Tracker	Precision scores	AUC scores
Ours	**0.716**	**0.513**
SiamAPN (coco)	0.699	0.486
TADT	0.687	0.508
SiamFC	0.680	0.473
UDT	0.575	0.430
AutoTrack	0.671	0.477
ARCF	0.666	0.473
CSRDCF	0.643	0.450
ECO_HC	0.640	0.468
STRCF	0.627	0.457
ARCF_F	0.612	0.434
MCCT_H	0.596	0.433
Staple_CA	0.578	0.420
SRDCF_decon	0.584	0.429
SRDCF	0.575	0.423
Staple	0.573	0.415
BACF	0.572	0.413
KCC	0.531	0.374
SAMF_CA	0.523	0.365
fDSST	0.516	0.379
SAMF	0.467	0.326
DSST	0.448	0.332
LCT	0.442	0.289
CN	0.434	0.287
DCF	0.408	0.266
KCF	0.406	0.265

#### Experiment on UAV20L dataset

**Fig 7:**
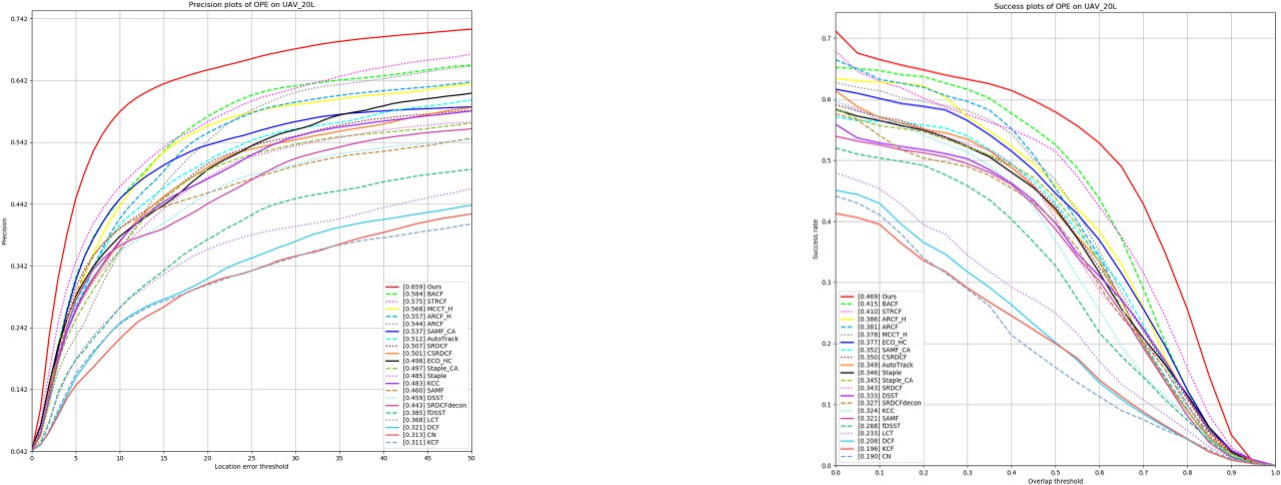
Experimental results on the UAV20L dataset.

The UAV20L dataset contains 20 long-duration tracking video sequences of over 2,000 frames each, totaling over 58,000 frames, covering a wide range of scenario designs and providing a basis for real-world tracking challenges. The dataset is suitable for evaluating tracker performance in long-duration tracking, especially in the face of target disappearance, reappearance, and long-term environmental changes. To validate the performance of our algorithm GAFFPFM in such scenarios, as shown in Table 2 and Fig 7, we compare it with 25 other trackers, including SiamAPN [9], TADT [32], SiamFC [23], UDT [33], AutoTrack [15], ARCF [34], ARCF_F [34], CSRDCF [35], ECO_HC [36], STRCF [17], MCCT_H [37], Staple [38], Staple_CA [38], SRDCF_decon [39], SRDCF [16], BACF [40], KCC, SAMF [41], SAMF_CA [41], fDSST [42], DSST [43], LCT [44], CN, DCF, and KCF [14]. These comparison algorithms include classical deep learning methods (e.g., SiamAPN, SiamFC, UDT, AutoTrack, etc.) as well as traditional filter-based tracking algorithms (e.g., ARCF, SRDCF, BACF, STRCF, KCF, etc.). The results show that our tracker performs superiorly in terms of both Precision and area under the curve (AUC). In the context of long-time tracking, our Precision improves by 6.4%, and our AUC score improves by 6.1%, compared to the baseline algorithm, SiamAPN, demonstrating the high performance of our algorithm in dealing with target disappearance and reappearance as well as long-term environmental changes.

**Table 2:** Experimental results on the UAV20L dataset of precision scores, success scores.

Tracker	Precision scores	AUC scores
Ours	**0.659**	**0.469**
SiamAPN (coco)	0.595	0.408
TADT	0.609	0.459
SiamFC	0.599	0.402
UDT	0.514	0.363
BACF	0.584	0.415
STRCF	0.575	0.410
MCCT_H	0.568	0.378
ARCF_H	0.557	0.386
ARCF	0.544	0.381
SAMF_CA	0.537	0.352
AutoTrack	0.512	0.349
SRDCF	0.507	0.343
CSRDCF	0.501	0.350
ECO_HC	0.498	0.377
Staple_CA	0.497	0.345
Staple	0.485	0.346
KCC	0.483	0.324
SAMF	0.460	0.321
DSST	0.459	0.333
SRDCF_decon	0.443	0.327
fDSST	0.385	0.288
LCT	0.368	0.233
DCF	0.321	0.208
CN	0.313	0.190
KCF	0.311	0.196

#### Experiment on DTB70 dataset

**Fig 8:**
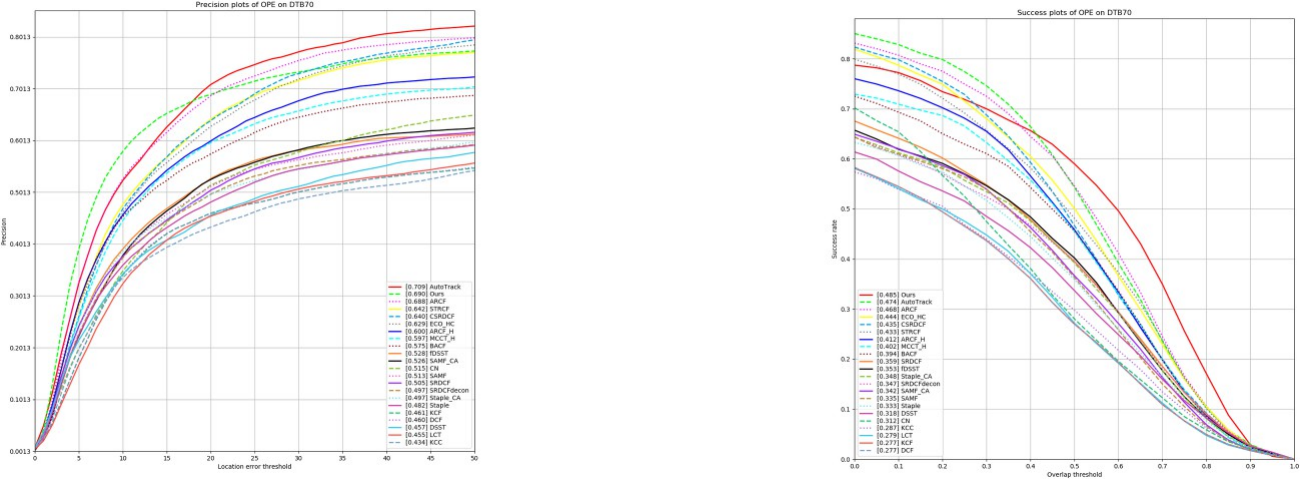
Experimental results on the DTB70 dataset.

The DTB70 dataset consists of 70 video sequences that feature targets and backgrounds of high complexity and challenge, including multiple factors interfering, diverse target classes, scene diversity, and long tracking times. Therefore, this dataset is particularly suitable for evaluating the performance of the tracker in the presence of complex backgrounds and interferences, testing the robustness of the tracking algorithms. As shown in Table 3 and Fig 8, we compare GAFFPFM with 25 other trackers, including SiamAPN [9], TADT [32], SiamFC [23], UDT [33], AutoTrack [15], ARCF [34], ARCF_F [34], CSRDCF [35], ECO_HC [36], STRCF [17], MCCT_H [37], Staple [38], Staple_CA [38], SRDCF_decon [39], SRDCF [16], BACF [40], KCC, SAMF [41], SAMF_CA [41], fDSST [42], DSST [43], LCT [44], CN, DCF, and KCF [14]. Compared to the baseline algorithm SiamAPN, our algorithm improves by 0.3% and 1% in Precision and AUC, respectively, showing the enhancement of tracker performance by perceptual feature modulation and feature refinement in complex contexts.

**Table 3:** Experimental results on the DTB070 dataset of precision scores, success scores.

Tracker	Precision scores	AUC scores
Ours	**0.690**	**0.485**
SiamAPN (coco)	0.687	0.475
TADT	0.693	0.464
SiamFC	0.735	0.487
UDT	0.602	0.422
AutoTrack	0.709	0.474
ARCF	0.688	0.468
STRCF	0.642	0.433
CSRDCF	0.640	0.435
ECO_HC	0.629	0.444
ARCF_H	0.600	0.412
MCCT_H	0.597	0.402
BACF	0.575	0.394
fDSST	0.528	0.353
SAMF_CA	0.526	0.342
CN	0.515	0.312
SAMF	0.513	0.335
SRDCF	0.505	0.359
SRDCF_decon	0.497	0.347
Staple_CA	0.497	0.348
Staple	0.482	0.333
KCF	0.461	0.277
DCF	0.460	0.277
DSST	0.460	0.318
LCT	0.455	0.279
KCC	0.434	0.287

Meanwhile, in order to compare our tracking speed, as shown in Table 4, in general, filtering-based algorithms have the advantage of fast-tracking speed; for example, KCF demonstrates its amazing tracking speed, but in deep learning-based, we demonstrate an amazing tracking speed of more than 100 frames in Table 4, which is ranked fifth in UAV123@10fps, UAV20L, and DTB70, and is a major breakthrough in deep learning, because we greatly improve as well as maintain the tracking speed based on the leading retention accuracy and AUC relative to the above algorithms. This is a major breakthrough in deep learning because compared to the above algorithms, our algorithm greatly improves and maintains the tracking speed on the basis of retaining the leading accuracy and AUC, i.e., it embodies the advancement of the anchorless frame mechanism, as well as the superiority of feature modulation and feature optimization in terms of detail capture and feature refinement, and proves the effectiveness of our algorithm in striking a balance between efficiency and accuracy.

**Table 4:** Comparison of tracking speed on UAV123@10pfs UAV20L and DTB70 by different algorithms.

Trackers	Dataset (fps/s)		
	UAV123_10fps	UAV_20L	DTB70
Ours	162.202	96.365	154.6
SiamAPN	172.87	130.66	167.96
Ocean	93.94	84.41	89.79
SESiam_FC	43.83	38.63	41.34
ARCF	23.30	23.14	24.32
STRCF	23.62	25.29	26.32
MCPF	0.53	0.56	0.61
ECO	16.70	11.94	11.61
MCCT	8.51	7.90	8.61
DeepSTRCF	5.74	5.83	6.20
AutoTrack	47.61	44.82	48.63
STRCF	22.40	17.41	21.88
MCPF	0.53	0.56	0.61
ECO	16.70	11.94	11.61
MCCT	8.51	7.90	8.61
DeepSTRCF	5.74	5.83	6.20
AutoTrack	47.61	44.82	48.63
CSRDCF	10.42	10.28	11.14
ECO_HC	58.11	62.63	54.67
ARCF_H	38.44	29.19	34.78
MCCT_H	46.37	41.16	48.91
BACF	37.81	32.00	36.37
fDSST	159.56	79.15	138.75
SAMF_CA	8.38	8.63	7.05
CN	308.80	203.91	198.05
SAMF	9.18	9.56	7.57
SRDCF	11.18	7.31	8.41
SRDCFdecon	5.44	3.98	4.18
Staple_CA	50.12	35.03	50.66
Staple	88.01	80.08	91.04
KCF	561.09	371.6	364.08
DCF	811.45	576.7	552.98
DSST	84.87	58.26	61.78
LCT	39.86	32.27	30.36
KCC	35.91	27.15	35.93

### Ablation experiments

**Fig 9:**
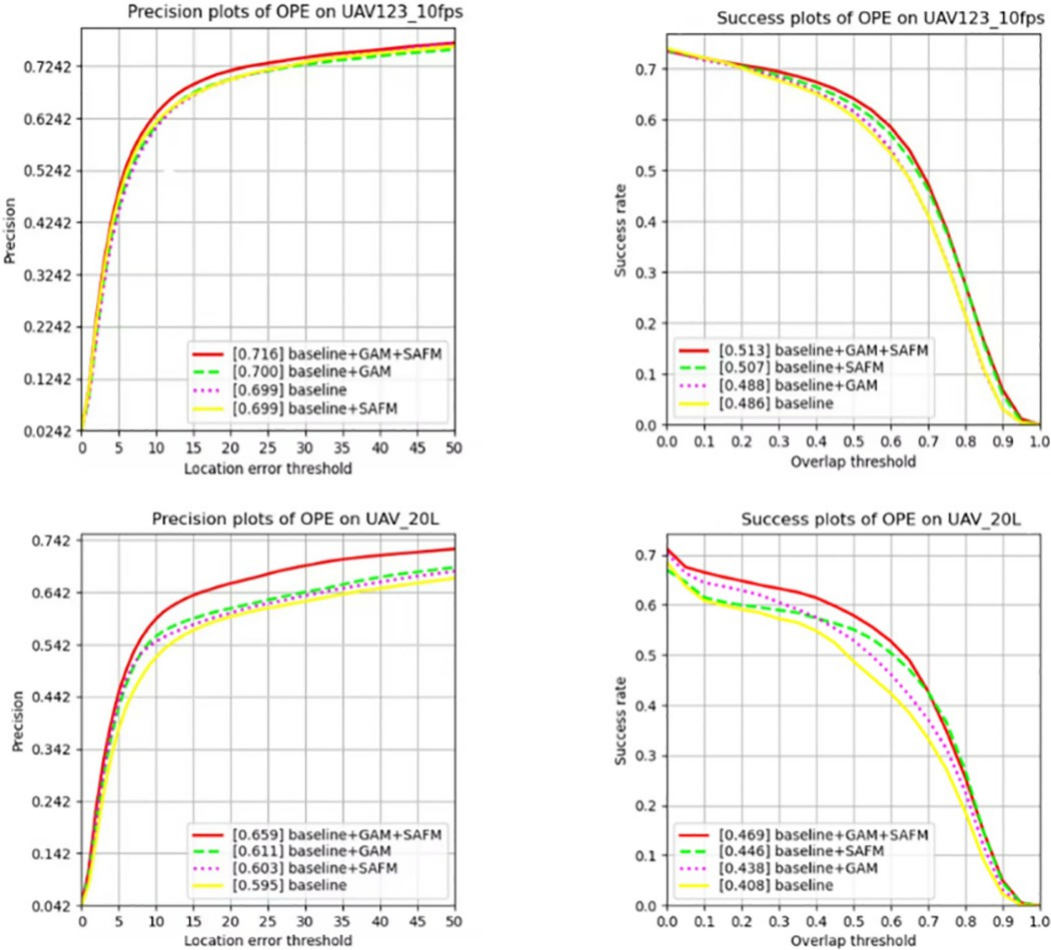
Experimental results on UAV20L and UAV123@10fps.

Table 5 and Fig 9 shows the comparison results under baseline, baseline + GAM, baseline + SAFM, and baseline + GAM + SAFM on the challenge datasets UAV123@10fps and UAV20L in UAV view. Under UAV123@10fps, when the baseline algorithm uses the coco dataset and is trained under the same conditions, the precision (P) and success rate (S) are 0.699 and 0.486, respectively. The baseline algorithm using global attention augmentation achieves a precision of 0.700 and a success rate of 0.488 under the same data and conditions. Meanwhile, the baseline with the SAFM improves the accuracy to 0.699 and the success rate to 0.507. When the two enhancement mechanisms are applied simultaneously, the tracking accuracy and the success rate are significantly enhanced to 0.716 and 0.513, respectively, as shown in the following table and figure. Similarly, the test results on the UAV20L dataset are displayed in the following table.

**Table 5:** Performance comparison of different combinations on UAV123@10FPS and UAV20L datasets.

Different module composition algorithms	UAV123@10FPS		UAV20L	
	P	S	P	S
Baseline (coco)	0.699	0.486	0.595	0.408
Baseline+GAM	0.700	0.488	0.611	0.438
Baseline+SAFM	0.699	0.507	0.603	0.446
Baseline+SAFM+GAM	**0.716**	**0.513**	**0.659**	**0.469**

### Qualitative comparison

**Fig 10:**
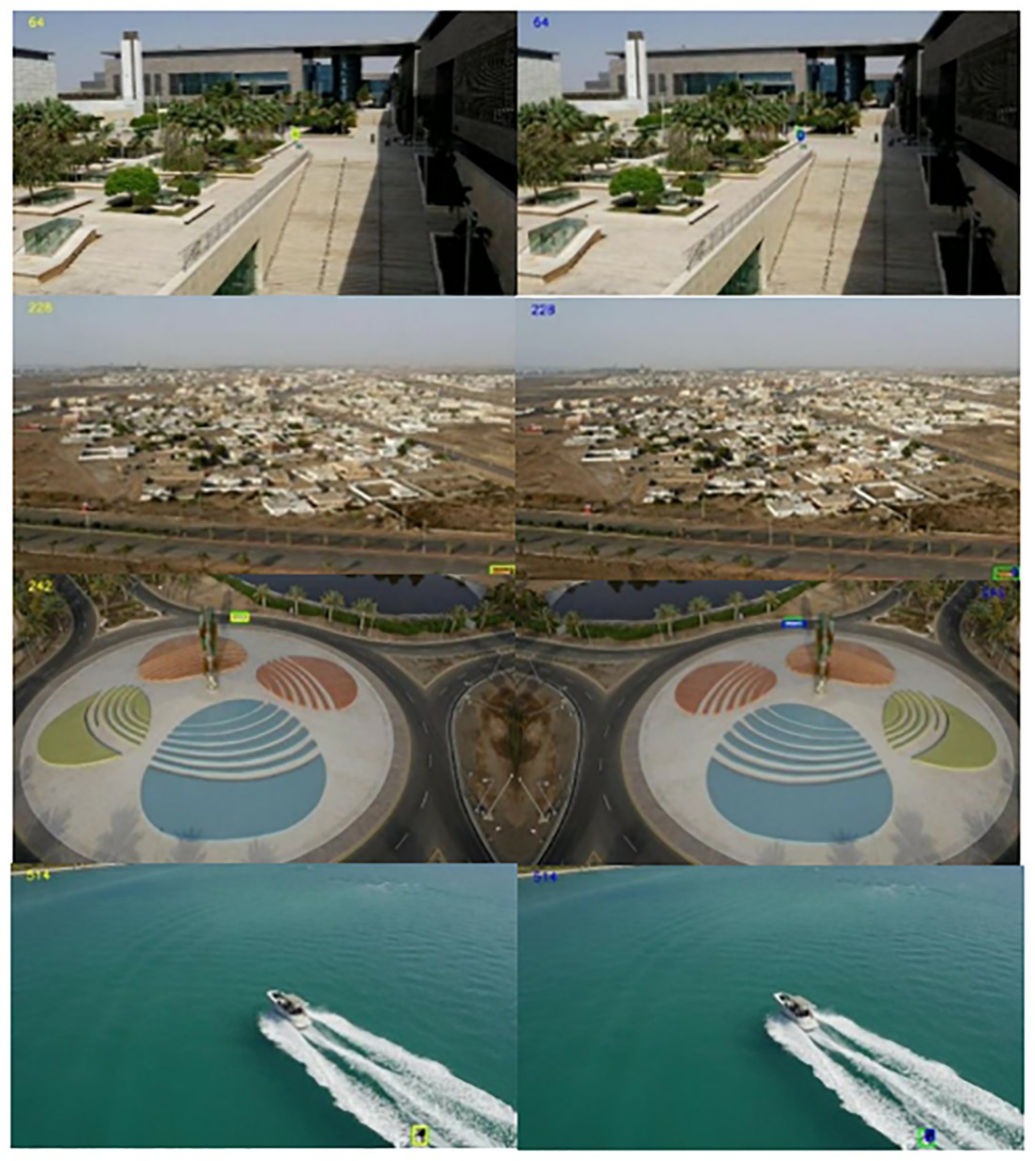
Comparison under UAV123@10fps dataset.

As shown in Fig 10, for comparison with the baseline, for the tracking effect under deep learning with the baseline algorithm, SiamAPN, we compared our algorithm with the Siamapn algorithm using the same training dataset on the UAV123@10fps dataset. The upper left corner represents the number of frames in the image: the yellow box indicates the prediction frame of our algorithm, the green box indicates the actual frame, and the blue box indicates the prediction frame of the Siampan algorithm. Our algorithm is closer to the range of the green prediction frame for pedestrian prediction (frame 64). For vehicle prediction, e.g., frame 228, the blue box becomes smaller and distorted when the vehicle is about to disappear from the field of view. In contrast, the yellow box of our algorithm still maintains a good overlap with the actual box. In addition, at frame 242 for the car and frame 514 for the swimmer’s rowing boat, our algorithm can accurately predict the target location and maintain overlap with the actual frame despite the significant magnitude changes in the upper and lower frames.

**Fig 11:**
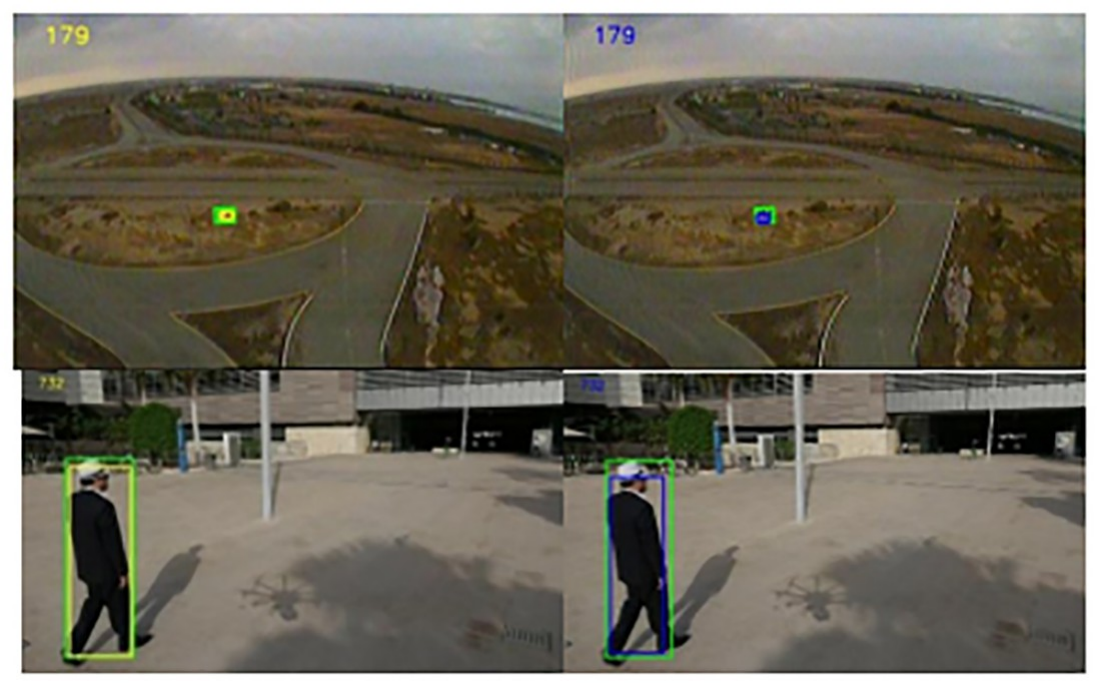
Comparison under UAV20L dataset.

As shown in Fig 11, two scenes selected from the UAV20L dataset (frames 179 and 732, respectively) demonstrate a small target car and a pedestrian walking in a dynamically changing viewpoint. The yellow frame in our algorithm follows the spatiotemporal transformation more accurately and is closer to the real frame.

### Physical verification

**Fig 12:**
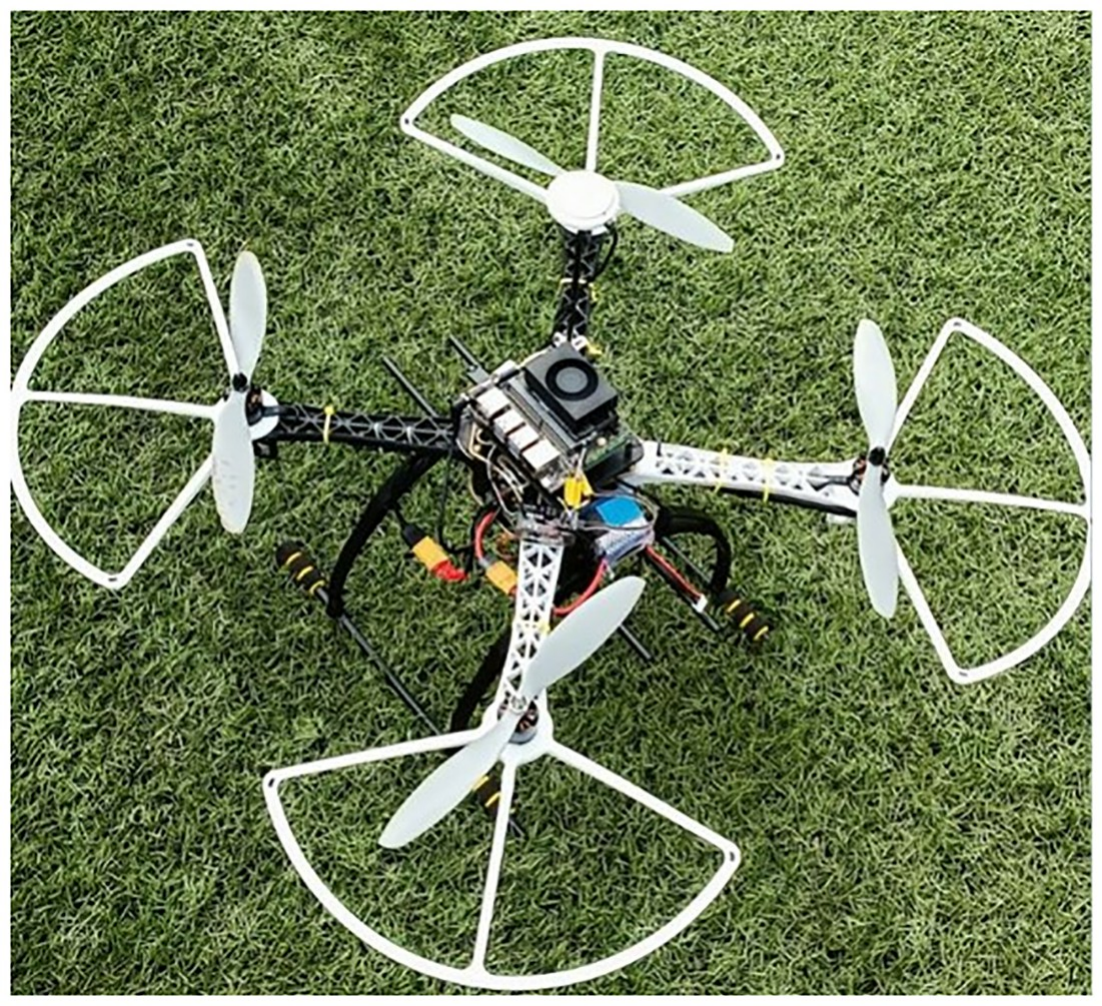
Physical drone.

**Fig 13:**
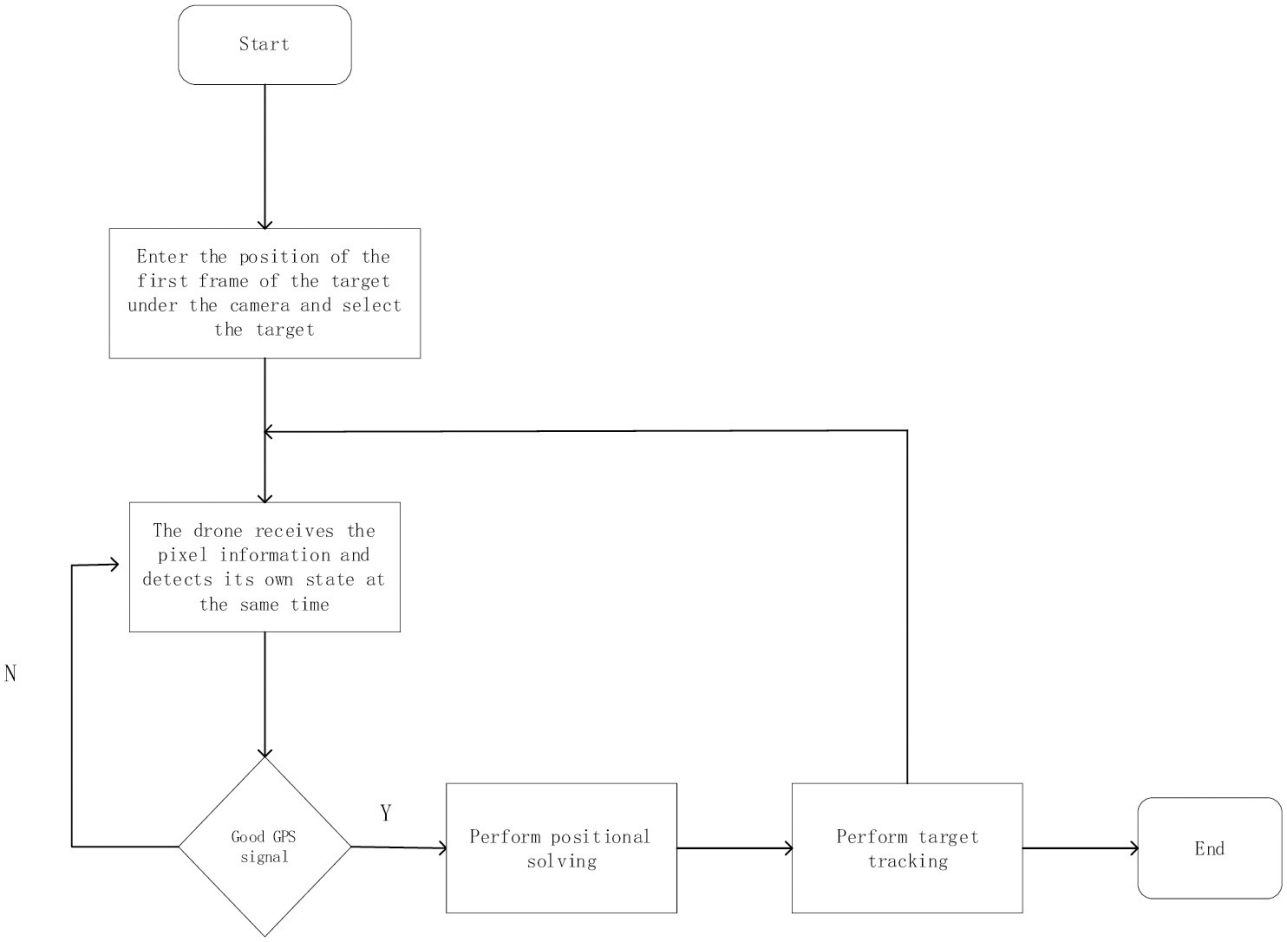
Flowchart of the drone takeoff system.

For this purpose, we built a complete UAV live system, as shown in Fig 12, for deploying and running our algorithms in real applications to verify their usability. The actual flow of the whole system is shown in Fig 13: First, the first frame of the target image for our algorithm is input. Then, the UAV will check its status to confirm whether it usually takes off and whether the target tracking algorithm runs normally. Subsequently, the system will select the target and frame it. The UAV acquires the pixel information, performs attitude solving to calculate the distance of the actual object from the target, and sends out speed control commands to fly toward the target. When the target position is updated, the UAV will adjust the flight direction while keeping the nose direction unchanged, adopting the headless mode to delineate the quadrant better and determine the exact position. The whole process is executed cyclically until the distance between the UAV and the target is less than a set threshold, indicating that the target has been reached directly above the target, and the tracking process then ends.

**Fig 14:**
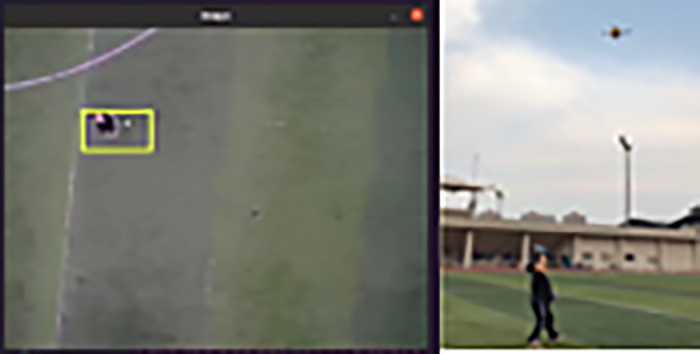
UAV target finding and target tracking.

**Fig 15:**
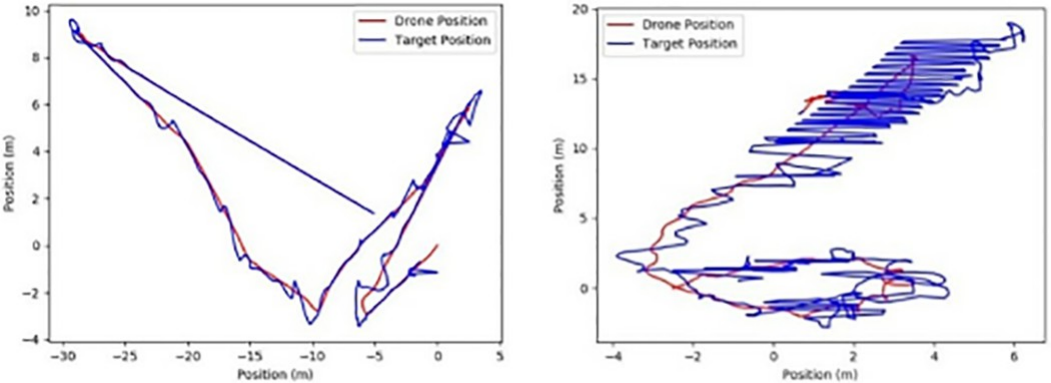
UAV target finding and target tracking.

The target is boxed after the UAV takes off, as shown in Fig 14 below; we have selected the target after the UAV takes off, thus realizing target acquisition and then target tracking. As shown in Fig 15, the motion trajectories of the UAV and the target object are shown separately under the simulation conditions. Their motion trajectories overlap. In the illustration, the red color indicates the position change of the UAV, and the blue color indicates the position change of the target. At the beginning of the virtual flight, we specify the direction of the nose of the UAV as the X-axis, the direction perpendicular to it as the Y-axis, and the takeoff point as the coordinate origin. In the coordinate axes in the figure, the horizontal axis is the X-axis, and the vertical axis is the Y-axis. As can be seen from the figure, the UAV recognizes the target after takeoff and follows towards it, and the trajectories of the two are coincident. As shown in Fig 15, the actual trajectory of the UAV and the target in the actual flight state is demonstrated in the physical condition. The curves are rather messy due to the frequent changes in the target’s position in the field of view caused by the changes in the UAV’s attitude during the actual flight. In the upper right corner of the figure, the UAV fails to follow the target accurately due to the rapid change of the target’s position and the UAV’s untimely response. However, after the takeoff point (0, 0), the UAV gradually follows the target and shows an overall trend of tracking the target.

## Conclusion

In this paper, a UAV target tracking method based on global feature interaction with anchorless frame-aware feature modulation is proposed. In the tracking subsystem, a channel space interaction mechanism is introduced to construct a real-time multi-scale feature modulation network of anchorless frame mechanisms for UAV target tracking with finer information representation and feature refinement. The P and S of the three datasets, UAV123@10fps, UAV20L, and DTB70, reached 0.716—0.659,0.5130—469,0.690—0.485, respectively. At the same time, we constructed a physical platform for UAV flights to validate the practical deployment and reliability of our algorithm. The experimental results show that the method performs well in terms of realism and effectiveness of target tracking. Later on, in terms of deep learning, we will also consider combining active learning methods with deep learning [45], aiming to optimize the training process of the deep learning tracking algorithm and improve the learning efficiency and performance of the model.

However, in the actual control subsystem, we found that the flight process of the UAV was not smooth enough, and the handling of target loss could be improved. In the future, we plan to optimize the smooth processing effect of target tracking by adjusting the PID parameters of tracking, including the attitude, angle, and speed control of the UAV. At the same time, we will employ Kalman filtering to predict the target’s position information, thus compensating for the time delay required for decision-making, transmission of coordinate points, and aircraft response to achieve synchronized tracking with the target. These improvements aim to design a complete UAV target tracking system to promote its relevance and effectiveness in practical applications.
